# Supplementary material for: Roles of two glutathione S-transferases in the final step of the β-aryl ether cleavage pathway in Sphingobium sp. strain SYK-6
Source: Sci Rep. 2020 Nov 26;10:20614. doi: 10.1038/s41598-020-77462-8 (PMC7691349; doi:10.1038/s41598-020-77462-8)
Supplement: Supplementary file 1 — Supplementary information. [file 41598_2020_77462_MOESM1_ESM.pdf]

Supplementary information

Roles of two glutathione *S*-transferases in the final step of the β-aryl ether cleavage pathway in *Sphingobium* sp. strain SYK-6

Yudai Higuchi[1,2], Daisuke Sato[1], Naofumi Kamimura[1] & Eiji Masai[1*]

[1]Department of Bioengineering, Nagaoka University of Technology, Nagaoka, Niigata 940-2188, Japan

[2]Present address: Faculty of Agriculture and Life Science, Hirosaki University, Hirosaki, Aomori 036-8561, Japan

*Corresponding author:
Eiji Masai
Department of Bioengineering, Nagaoka University of Technology, Nagaoka, Niigata 940-2188, Japan
E-mail: emasai@vos.nagaokaut.ac.jp
TEL: +81 258 47 9428
ORCID: 0000-0001-9194-7483

Contents list

Supplementary tables: Table S1-S4
Supplementary figures: Fig. S1-S12
References for Supplementary information

Table S1. Enzyme properties of LigG and its related enzymes

Strain	Enzyme	Substrate	Stereo-specificity	Sp act[a] ($\mu mol \cdot min^{-1} \cdot mg^{-1}$)	k_{cat} (s^{-1})	$K_{m,}$ GS-HPV (μM)	$K_{m,}$ GSH (mM)	$k_{cat}/K_{m,}$ GS-HPV ($mM^{-1} \cdot s^{-1}$)	Rate *R*/ Rate *S*[b]	Reference
Sphingobium sp. SYK-6	LigG	GS-HPV	β*R*	33 ± 1			1.19 ± 0.22		300	This study
			β*S*	0.11 ± 0.01						
	LigG	GS-HPV	β*R*	80 ± 9	28 ± 1	16 ± 1		1700 ± 170		1
			β*S*							
	LigG	GS-βVG[c]	β*R*	1700					18000	2
			β*S*	0.094						
Novosphingobium sp. PP1Y	NsLigG	GS-βVG	β*R*	71					3200	2
			β*S*	0.022						
Thiobacillus denitrificans ATCC25259	TdLigG	GS-βVG	β*R*	36					300	2
			β*S*	0.12						

[a]Specific activity

[b]The values were obtained by dividing the specific activity for (β*R*)-GS-HPV derivatives by the specific activity for (β*S*)-GS-HPV derivatives.

[c]β-*S*-glutathionyl-α-veratrylglycerone

Table S2. Enzyme properties of LigQ and its related enzymes

Strain	Enzyme	Substrate	Stereo-specificity	Sp act[a] (μmol·min^{-1}·mg^{-1})	k_{cat} (s^{-1})	$K_{m,\ GS\text{-}HPV}$ (μM)	$K_{m,\ GSH}$ (mM)	$k_{cat}/K_{m,\ GS\text{-}HPV}$ (mM^{-1}·s^{-1})	Rate *S*/ Rate *R*	Reference
Sphingobium sp. SYK-6	LigQ	GS-HPV	β*R*	17 ± 2			0.34 ± 0.10		6[b]	This study
			β*S*	110 ± 14						
	LigQ	GS-HPV	β*R*		13 ± 1	55 ± 7		240 ± 40	11[c]	3
			β*S*		30 ± 5	11 ± 2		2700 ± 700		
Novosphingobium aromaticivorans DSM 12444	$NaGST_{Nu}$	GS-HPV	β*R*		80 ± 10	40 ± 6		1900 ± 400	4[c]	3
			β*S*		57 ± 9	8 ± 3		8000 ± 3000		
Novosphingobium sp. MBES04	NmGST3	GS-HPV	β*R*	3.9					2[b]	4
			β*S*	9.1						
Escherichia coli DH5α	YghU	GS-HPV	β*R*		0.43 ± 0.03	28 ± 4		16 ± 3	1.5[c]	3
			β*S*		0.29 ± 0.03	12 ± 3		24 ± 6		
	YfcG	GS-HPV	β*R*		0.04 ± 0.01	160 ± 60		0.2 ± 0.1	0.7[c]	3
			β*S*		0.017 ± 0.004	130 ± 40		0.14 ± 0.06		

[a]Specific activity

[b]The values were obtained by dividing the specific activity for (β*S*)-GS-HPV by the specific activity for (β*R*)-GS-HPV.

[c]The values were obtained by dividing k_{cat}/K_m for (β*S*)-GS-HPV by k_{cat}/K_m for (β*R*)-GS-HPV.

Table S3. Strains and plasmids used in this study

Strain or plasmid	Relevant characteristic(s)[a]	Reference or source
Strains		
Sphingobium sp.		
SYK-6	Wild type; Nal^r Sm^r	5
Δ*ligQ* (SME226)	SYK-6 derivative; Δ*ligQ*; Nal^r Sm^r	This study
Δ*ligG* (SME273)	SYK-6 derivative; Δ*ligG*; Nal^r Sm^r	This study
Δ*ligG ligQ* (SME274)	SYK-6 derivative; Δ*ligG ligQ*; Nal^r Sm^r	This study
Escherichia coli		
BL21(DE3)	F^- *ompT hsdS*$_B$(r_B^- m_B^-) *gal dcm* (DE3); T7 RNA polymerase gene under the control of the *lacUV5* promoter	6
HB101	*recA13 supE44 hsd20 ara-14 proA2 lacY1 galK2 rpsL20 xyl-5 mtl-1*	7
NEB 10-beta	Δ(*ara-leu*) *7697 araD139 fhuA* Δ*lacX74 galK16 galE15 e14-* ϕ*80*Δ*lacZ*Δ*M15 recA1 relA1 endA1 nupG rpsL* (Sm^r) *rph spoT1* Δ(*mrr-hsdRMS-mcrBC*)	New England Biolabs
Plasmids		
pRK2013	Tra^+ Mob^+ ColE1 replicon; Km^r	8
pET-16b	Expression vector; T7 promoter, Ap^r	Novagen
pAK405	Plasmid for allelic exchange and markerless gene deletions in Sphingomonads; Km^r	9
pQF	Expression vector; P_{Q5} high-GC-content codon-optimized *cymR*, Tc^r	10
pET00360	pET-16b with a 0.7-kb NdeI-BamHI PCR amplified fragment carrying SLG_00360	This study
pET04120	pET-16b with a 0.9-kb NdeI-BamHI PCR amplified fragment carrying *ligQ*	This study
pET08650	pET-16b with a 0.8-kb NdeI-BamHI PCR amplified fragment carrying *ligF*	This study
pET08660	pET-16b with a 0.9-kb NdeI-BamHI PCR amplified fragment carrying *ligE*	This study
pET08670	pET-16b with a 0.8-kb NdeI-BamHI PCR amplified fragment carrying *ligG*	This study
pET29340	pET-16b with a 0.7-kb NdeI-BamHI PCR amplified fragment carrying SLG_29340	This study
pAK04120	pAK405 with a 2.8-kb deletion cassette carrying up- and downstream regions of *ligQ*	This study
pAK08670	pAK405 with a 2.1-kb deletion cassette carrying up- and downstream regions of *ligG*	This study
pQF*ligQ*	pQF with a 0.9-kb PCR amplified fragment carrying *ligQ*	This study
pQF*ligG*	pQF with a 0.8-kb PCR amplified fragment carrying *ligG*	This study

[a]Nal^r, Sm^r, Km^r, Tc^r, and Ap^r, resistance to nalidixic acid, streptomycin, kanamycin, tetracycline, and ampicillin, respectively.

Table S4. Primers used in this study

Plasmid or strain	Primer	Sequence (5′ to 3′)
Construction of plasmids		
pET00360	pET00360_F	TCGAAGGTCGTCATATGCTCCGGCTCATCATC
	pET00360_R	GTTAGCAGCCGGATCCTCAGCCCCGCGGAGCGGC
pET04120	pET04120_F	TCGAAGGTCGTCATATGGCCGACAGCGACCCCT
	pET04120_R	GTTAGCAGCCGGATCCTCAGGCCTCGGTCTTCGC
pET08650	pET08650_F	TCGAAGGTCGTCATATGACGTTGAAACTCTACAG
	pET08650_R	GTTAGCAGCCGGATCCTCAGGCGACTTTCTCGTTCT
pET08660	pET08660_F	TCGAAGGTCGTCATATGGCCAGGAACAACACCAT
	pET08660_R	GTTAGCAGCCGGATCCTCAGTCCGCTTTCTCGGCGA
pET08670	pET08670_F	TCGAAGGTCGTCATATGGCCGAGCCACAGGAAC
	pET08670_R	GTTAGCAGCCGGATCCTCAGCGGGTGAGGCCCAGT
pET29340	pET29340_F	TCGAAGGTCGTCATATGTGGCACCTCTATCAATT
	pET29340_R	GTTAGCAGCCGGATCCTCAGAAATCCGGCTGGTC
pAK04120	dis04120_top_F	CGGTACCCGGGGATCAGCCAGAACCTCTACAAGCG
	dis04120_top_R	AATAGAGCTGGATGGGGTGC
	dis04120_bot_F	CGACTCTAGAGGATCCATCGGCGGGGAATATAGCG
	dis04120_bot_R	GCACCCCATCCAGCTCTATTGTGCTGGACAAGAACCTGGC
pAK08670	dis08670_top_F	CGGTACCCGGGGATCAGCGGCAGAACGAGAAAGTC
	dis08670_top_R	CATGATTTCCACACGCTCGG
	dis08670_bot_F	CGACTCTAGAGGATCCGACCCGCTGCTACTTGTTG
	dis08670_bot_R	CCGAGCGTGTGGAAATCATGATTATGACTACACGCAGGGC
pQF*ligQ*	pQF*ligQ*_F	CTAGTAGAGGAAGCTATGGCCGACAGCGACCCCTC
	pQF*ligQ*_R	TCACTTCACCGGATCTCAGGCCTCGGTCTTCGCGG
pQF*ligG*	pQF*ligG*_F	CTAGTAGAGGAAGCTATGGCCGAGCCACAGGAACT
	pQF*ligG*_R	TCACTTCACCGGATCTCAGCGGGTGAGGCCCAGTT
Colony PCR		
Δ*ligG*	dis08670_conf_F	CAATTTCGCCATCGCCAATG
	dis08670_bot_R	CCGAGCGTGTGGAAATCATGATTATGACTACACGCAGGGC
Δ*ligQ*	dis04120_top_F	CGGTACCCGGGGATCAGCCAGAACCTCTACAAGCG
	dis04120_conf_R	CGGTACCCGGGGATCTGCATGCGGGCATTGAGATC

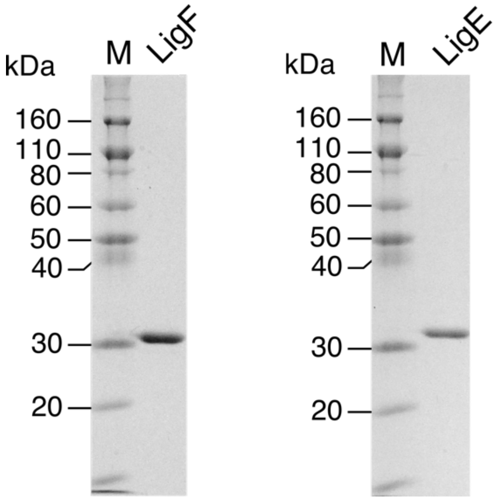

Fig. S1. LigF and LigE purification.

Proteins were separated on SDS-12% polyacrylamide gels and stained with Coomassie Brilliant Blue. Lanes: LigF, Purified LigF (1 µg) from *E. coli* BL21(DE3) harboring pET08650; LigE, Purified LigE (1 µg) from *E. coli* BL21(DE3) harboring pET08660; M, molecular mass markers. The predicted molecular masses of His-tag-fused LigF and LigE are 32,206 and 34,591 Da, respectively. The cropped gel images are shown, and the full-length gel is presented in Fig. S12.

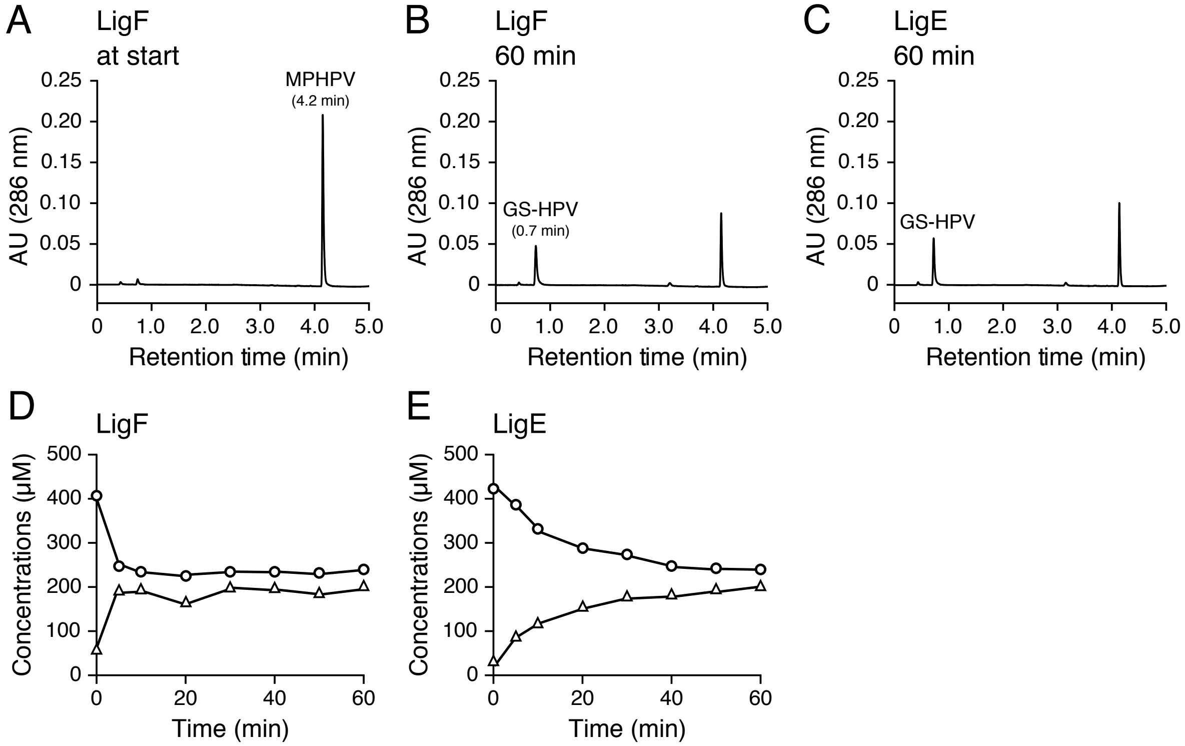

Fig. S2. Conversion of MPHPV into GS-HPV by purified LigF and LigE.

MPHPV (400 μM) was incubated with LigF (A, B, and D; 40 μg protein/ml) and LigE (C and E; 150 μg protein/ml) in the presence of 5 mM GSH. Portions of the reaction mixtures were collected, and the amount of MPHPV (circles) and GS-HPV (triangles) were measured using HPLC. (A and B) HPLC chromatograms of the reaction mixtures incubated with LigF at the start and after 60 min, respectively. (C) HPLC chromatogram of the reaction mixture incubated with LigE after 60 min. (D and E) Time course of MPHPV conversion to GS-HPV by LigF and LigE, respectively.

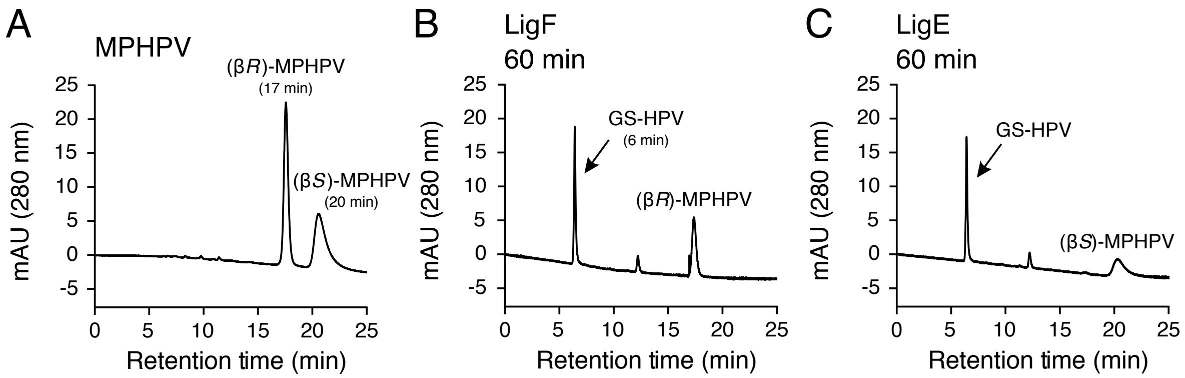

Fig. S3. Chiral HPLC analysis of MPHPV conversion by LigF and LigE.

(A) Chiral HPLC chromatogram of the racemic MPHPV. MPHPV (400 µM) was incubated with LigF (B; 40 µg protein/ml) and LigE (C; 150 µg protein/ml) in the presence of 5 mM GSH for 60 min and then analyzed through chiral HPLC.

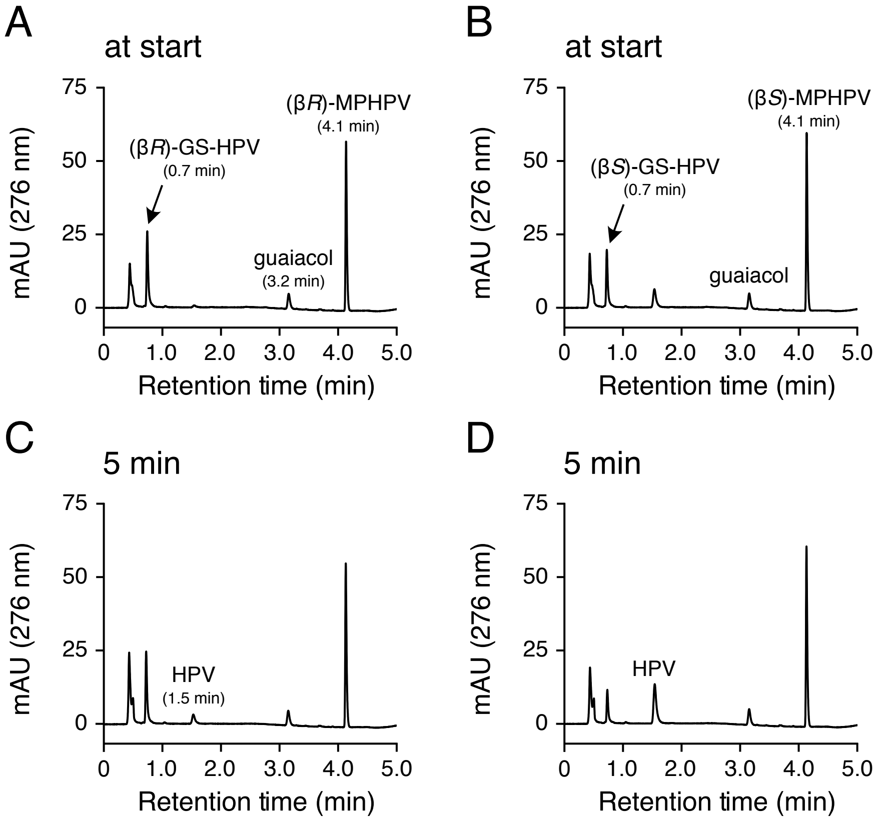

Fig. S4. HPLC analysis of GS-HPV metabolites.

Cell extract of SYK-6 (200 µg protein/ml) was incubated with 100 µM (β*R*)-GS-HPV (A and C) and (β*S*)-GS-HPV (B and D) in the presence of 2.4 mM GSH, respectively. Portions of the reaction mixtures were collected at the start (A and B) and after 5 min (C and D) of incubation and then analyzed through HPLC.

```
SLG_00360          ------------------MLRLIIGNKVYSSWSLRGWLAAKLSG------LPFEEVVVPL   36
SLG_04120(LigQ)    PIQLYSL---G------------------TPNGQKVTIMLEELLAAGFD-AEYDAWLIKI   89
SLG_06390          ILHDYWR----------------------SGASYRVRIALNIKG------LAYEQVAHDL   39
SLG_07230          AVDLYHG--EP------------------GSNSLKVLQAIHEKG------VPFTSHYINL   35
SLG_08650(LigF)    TLKLYSF--GP------------------GANSLKPLATLYEKG------LEFEQVFVDP   35
SLG_08660(LigE)    TITLYDLQLES--------------GCTISPYVWRTKYALKHKG------FDIDIVPGGF   45
SLG_08670(LigG)    ELTIYHIPG--------------------CPFSERVEIMLELKG------LR--MKDVEI   37
SLG_11530          ------------------------------------------------------------   0
SLG_24810          ---------------------MIIVHHLDNSRSQRVLWLLEELG------LPYEIRRYQR   33
SLG_28090          -MKLHWGKM--------------------SPFARKVMVTAHETG------TDGRIELIDT   33
SLG_28330          -MKLYDRQG--------------------TPNAARIRIVLAEKG------LEDQVEFVTV   33
SLG_29340          MWHLYQFPL--------------------CPFSRKLRFLMAEKG------IVYELVRESP   34
SLG_31200          -MRLHWSPR--------------------SPYVRKVVILAHELG------LYSRIDRVRS   33
SLG_32600(LigP)    KITIYDLALAS--------------GATISPFVWATKYAIAHKG------FELDIVPGGF   45
SLG_33010          ILHEYAP----------------------SGNCYKIRLTAALLG------LPLARRHYDI   37
SLG_33060          MTAAIQFYTNP------------------MSRGQIIRWMLEELG------QPYETHLLDY   36
SLG_34480          RFHLYVSYACPWAHRALIVRALKGLDDMIGVSVVDPL--MREHG------WTFETARGGT   105
SLG_36080          TYQLYAHPF--------------------SSYCWKAMIAFHEKD------LHYELEMVEN   35
NmGST3             MLELWTS---E------------------TPNGWKTTIMLEELD------ANYTLRPISL   33
NmGST4             MLTLYSF--GP------------------GANSLKPLLALYEKG------LEFTPRFVDP   34
NmGST5             RITLYDLQLAS--------------GCTISPFVWRTKYALAHKG------FDMDIVPGGF   45
NmGST6             ALRMYQIPG--------------------CPFSERVELLLDLKG------LGDVLVDHEI   44
NsLigF             MLTLYSF--GP------------------GANSLKPLLALYEKG------LEFTPRFVDP   34
NsLigE             RITLYDLQLAS--------------GCTISPFVWRTKYALAHKG------FDVDIVPGGF   45
NsLigG             ALRMYHIPG--------------------CPFSERVEILLDLKG------LSGIMDDHEV   44
NaLigF1            MLKLYSF--GP------------------AANSMKPLLTVFEKG------LDVEKHRLDP   45
NaLigF2            ALKYYHA--EP------------------LANSLKSMVPLKEKG------LAYESIYVDL   35
NaLigE             TITFYDLALST--------------GATISPFVWATKYALKHKG------FDLDVVPGGF   45
NaGSTNu            PFQVYSL---G------------------TPNGQKATIMLEELLQLGFSEAEYDAWLIKI   81
TdLigG             RPTVYHIPV--------------------CPFCQRVEILLSLKG------RREDVDFRMI   36
```

Fig. S5. Amino acid sequence alignment of SYK-6 GSTs with other bacterial GSTs involved in β-aryl ether conversion.

Putative catalytic residues of *omega*-class GSTs located in the N-terminal region are shown with a black background.

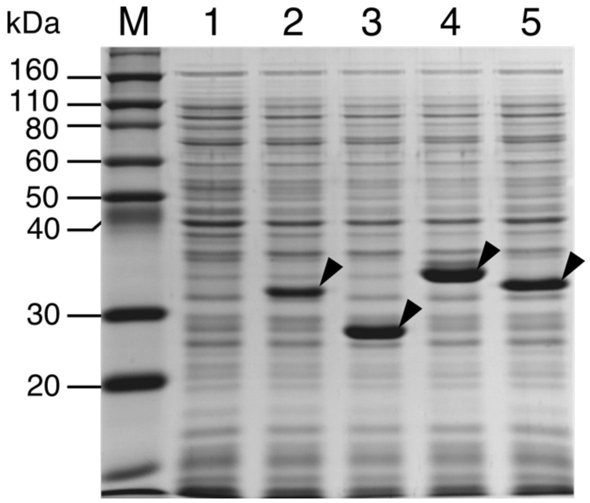

Fig. S6. SYK-6 GST gene expression in *E. coli*.

Cell extracts (10 μg) of *E. coli* BL21(DE3) carrying each of SLG_08670 (*ligG*), SLG_00360, SLG_04120, and SLG_29340 were separated by SDS-12% polyacrylamide gels and stained with Coomassie Brilliant Blue. Lanes: 1, *E. coli* BL21(DE3) harboring pET-16b; 2, *E. coli* BL21(DE3) harboring pET08670; 3, *E. coli* BL21(DE3) harboring pET00360; 4, *E. coli* BL21(DE3) harboring pET04120; 5, *E. coli* BL21(DE3) harboring pET29340; M, molecular mass markers. The predicted molecular masses of His-tag-fused gene products of SLG_08670 (*ligG*), SLG_00360, SLG_04120, and SLG_29340 are 32,800, 27,864, 35,776, and 27,960 Da, respectively. The SLG_29340 gene product showed a size somewhat larger (ca. 33 kDa) than the molecular mass calculated from the deduced amino acid sequence (27,960 Da); however, the nucleotide sequencing confirmed that SLG_29340 was correctly cloned in pET29340. The cropped gel image is shown, and the full-length gel is presented in Fig. S12.

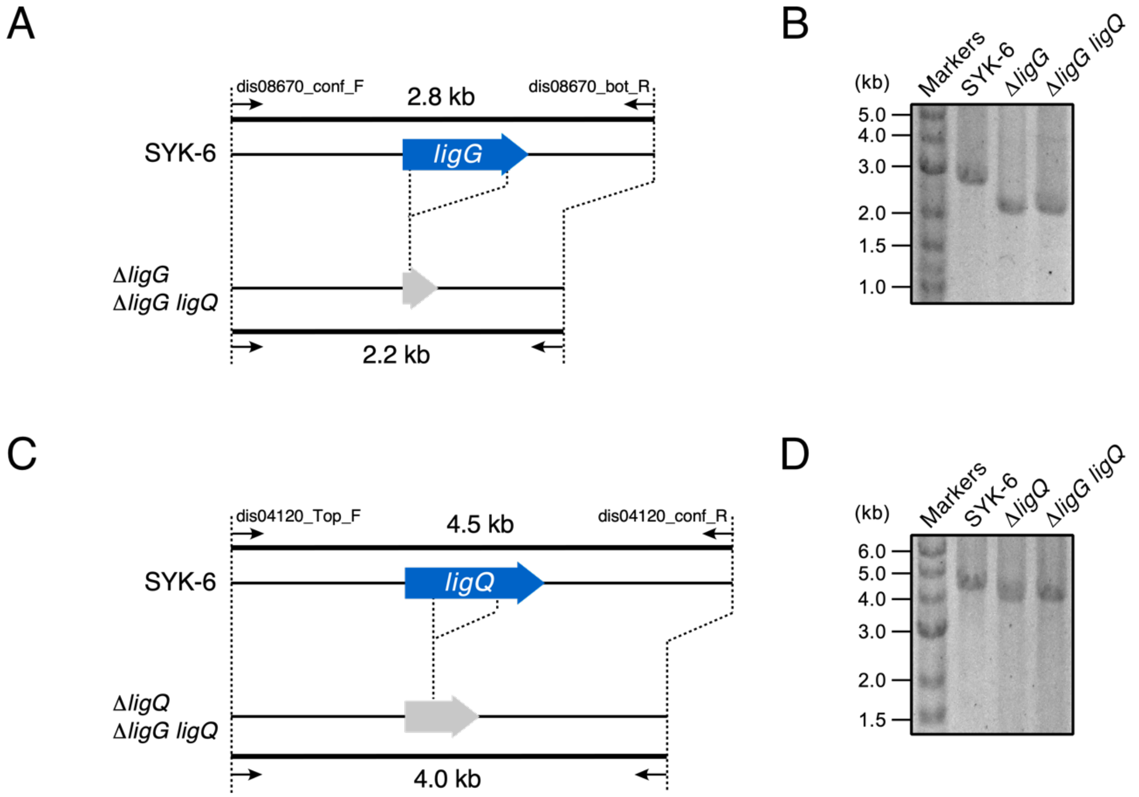

Fig. S7. Disruption of *ligG* and *ligQ* in SYK-6.

Schematic representations of *ligG* (A) and SLG_04120 (*ligQ*; C) disruption through homologous recombination. (B and D) Colony PCR analysis of the mutants using primer pairs of dis08670_conf_F–dis08670_bot_R (B) and dis04120_top_F–dis04120_conf_R (D) (Table S4). The cropped gel images are shown, and the full-length gel is presented in Fig. S12.

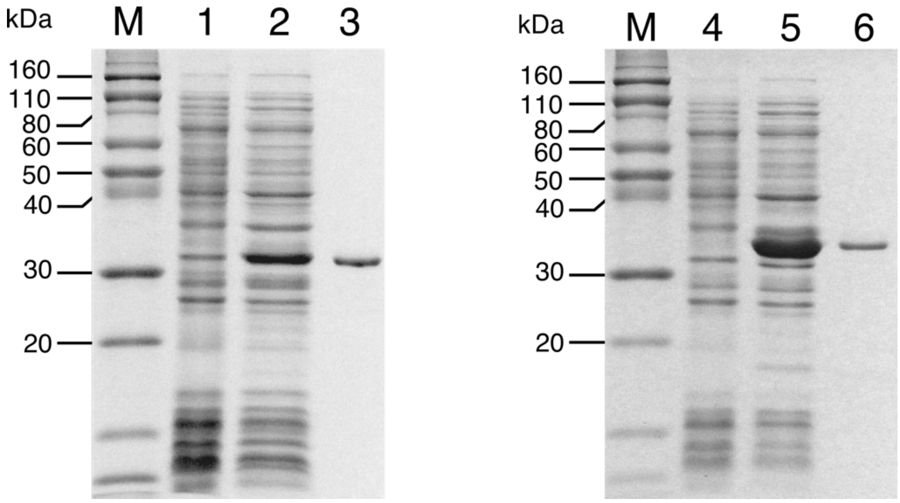

Fig. S8. LigG and LigQ purification.

The proteins were separated on SDS-12% polyacrylamide gels and stained with Coomassie Brilliant Blue. Lanes: 1 and 4, cell extract of *E. coli* BL21(DE3) harboring pET-16b (10 μg); 2, cell extract of *E. coli* BL21(DE3) harboring pET08670 (*ligG*, 10 μg); 3, purified LigG (1 μg); 5, cell extract of *E. coli* BL21(DE3) harboring pET04120 (*ligQ*, 10 μg); 6, purified LigQ (1 μg); M, molecular mass markers. The cropped gel images are shown, and the full-length gels are presented in Fig. S12.

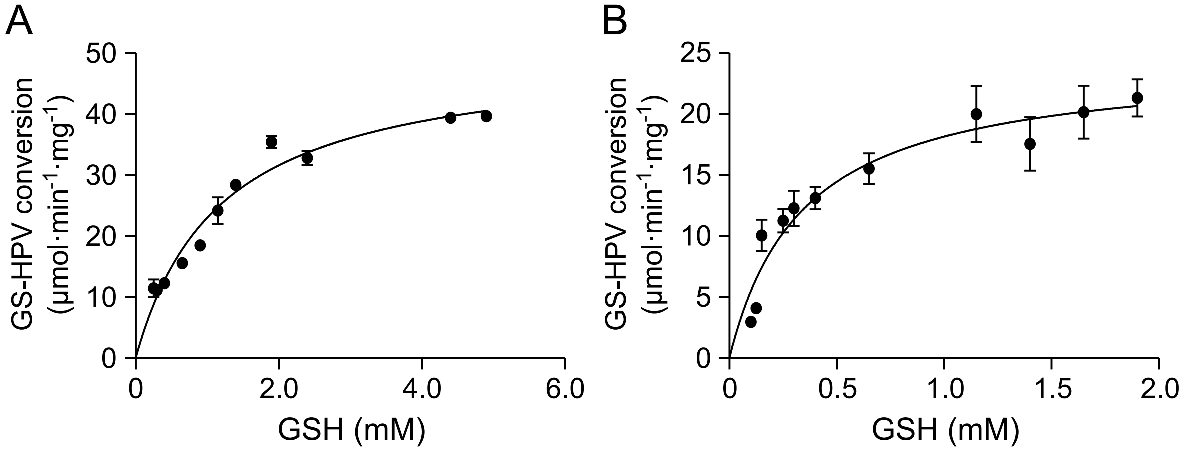

Fig. S9. LigG (A) and LigQ (B) activities measured at various GSH concentrations.

The reactions were conducted in 50 mM Tris-HCl buffer (pH 7.5) containing 100 μM (β*R*)-GS-HPV and GSH (0.25–4.9 mM for LigG and 0.1–1.9 mM for LigQ) at 30°C. All experiments were conducted in triplicate, and each value represents the mean ± standard deviation. K_m was calculated through non-linear regression analysis using the GraphPad Prism 7 software (GraphPad Software Inc.) fitted to the Michaelis–Menten equation.

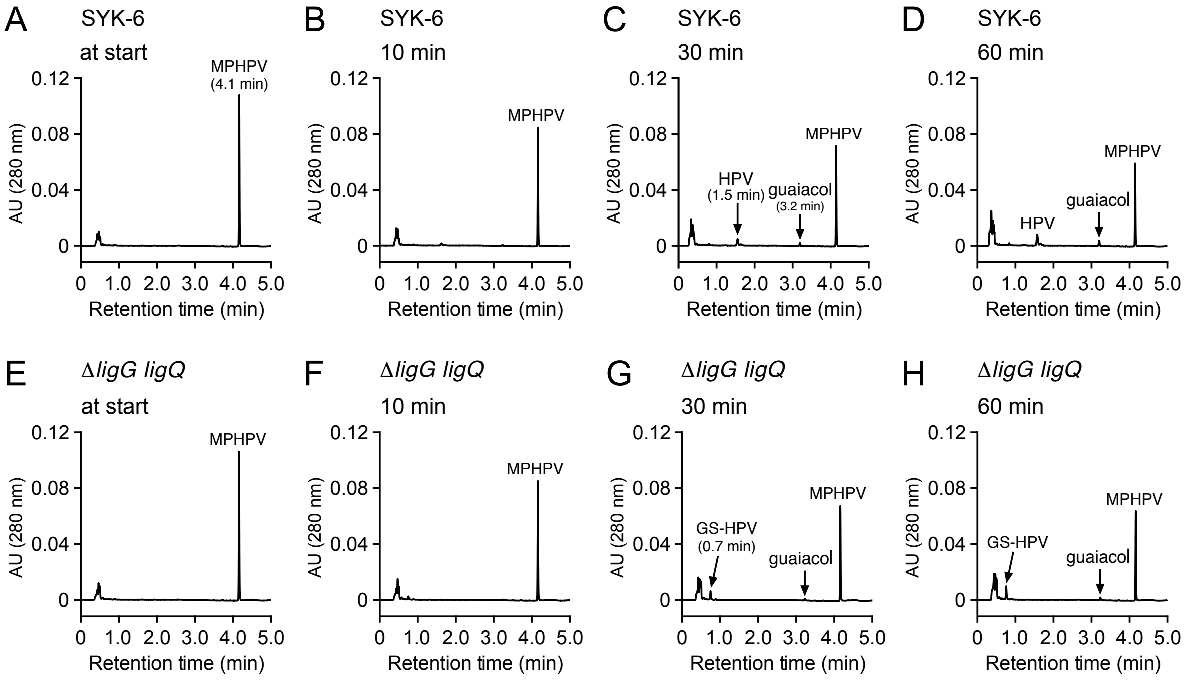

Fig. S10. Conversion of MPHPV by cell extracts of SYK-6 and Δ*ligG ligQ*.

Racemic MPHPV (200 μM) was incubated with cell extracts (1 mg protein/ml) of SYK-6 (A–D) and Δ*ligG ligQ* (E–H) in the presence of 5.0 mM GSH, respectively. Portions of the reaction mixtures were collected at the start (A and E) and after incubation for 10 min (B and F), 30 min (C and G), and 60 min (D and H), respectively, and then analyzed through HPLC.

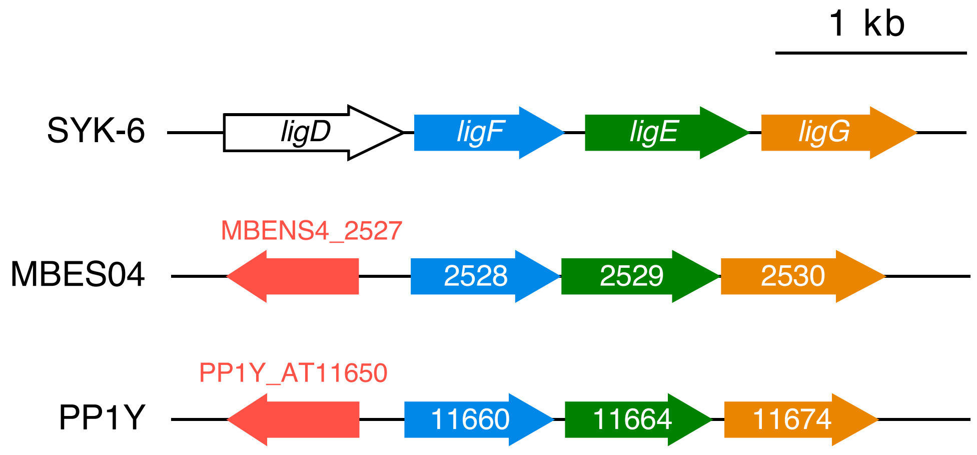

Fig. S11. Gene organization of the β-aryl ether cleavage pathway genes in Sphingomonads.
Genes: *Sphingobium* sp. SYK-6: *ligD* (SLG_08640), *ligF* (SLG_08650), *ligE* (SLG_08660), and *ligG* (SLG_08670). *Novosphingobium* sp. MBES04: MBENS4_2527 (NmGST3), MBENS4_2528 (NmGST4), MBENS4_2529 (NmGST5), and MBENS4_2530 (NmGST6). *Novosphingobium* sp. PP1Y: PP1Y_AT11650, PP1Y_AT11660 (NsLigF), PP1Y_AT11664 (NsLigE), and PP1Y_AT11674 (NsLigG).

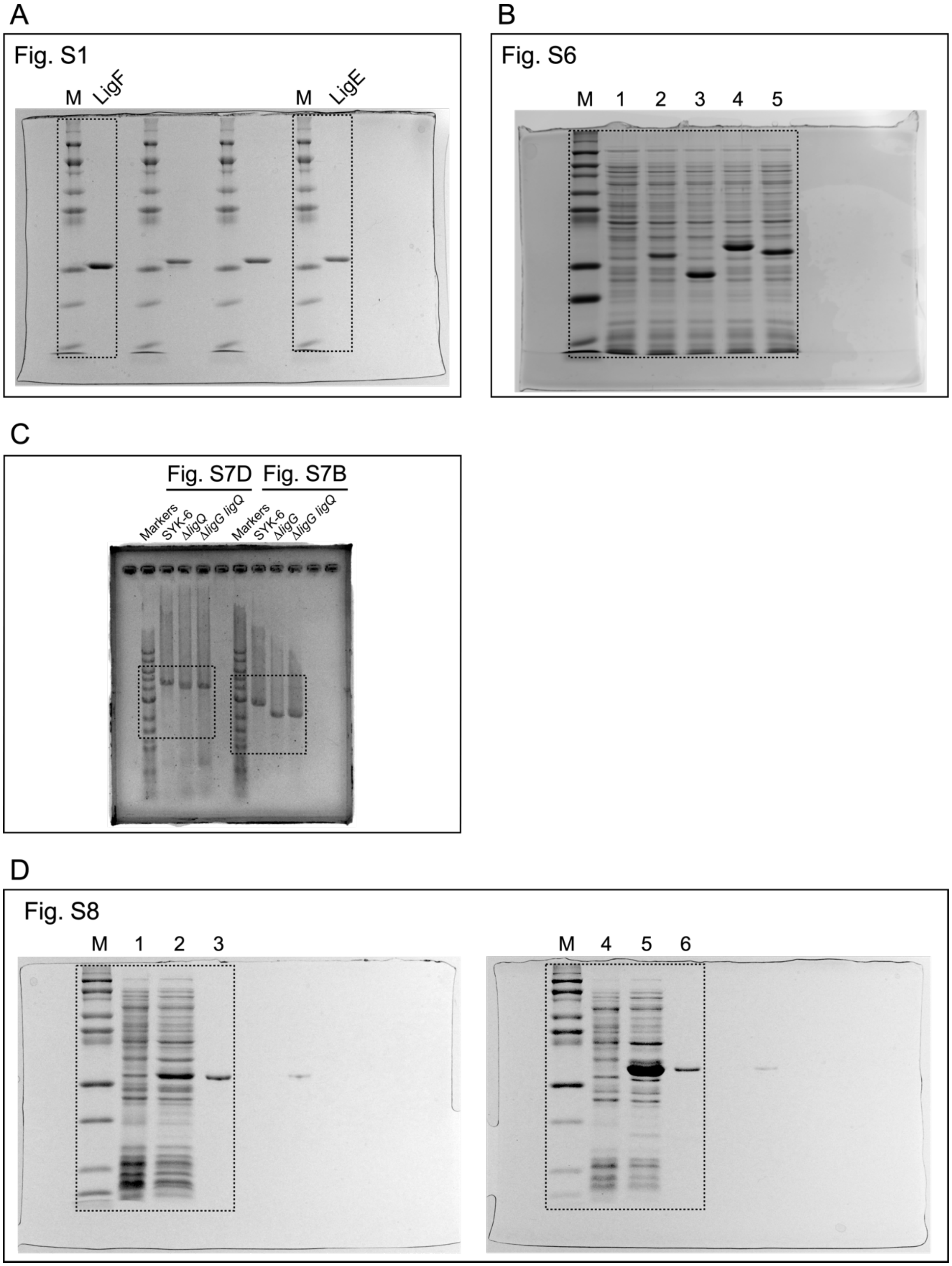

Fig. S12. Uncropped gel images shown in Fig. S1, Fig. S6, Fig. S7, and Fig. S8.

References

1 Pereira, J. H. *et al.* Structural and biochemical characterization of the early and late enzymes in the lignin β-aryl ether cleavage pathway from *Sphingobium* sp. SYK-6. *J. Biol. Chem.* **291**, 10228-10238, doi:10.1074/jbc.M115.700427 (2016).

2 Picart, P., Sevenich, M., de María, P. D. & Schallmey, A. Exploring glutathione lyases as biocatalysts: paving the way for enzymatic lignin depolymerization and future stereoselective applications. *Green Chem.* **17**, 4931-4940, doi:10.1039/c5gc01078k (2015).

3 Kontur, W. S. *et al. Novosphingobium aromaticivorans* uses a Nu-class glutathione *S*-transferase as a glutathione lyase in breaking the β-aryl ether bond of lignin. *J. Biol. Chem.* **293**, 4955-4968, doi:10.1074/jbc.RA117.001268 (2018).

4 Ohta, Y. *et al.* Enzymatic specific production and chemical functionalization of phenylpropanone platform monomers from lignin. *ChemSusChem* **10**, 425-433, doi:10.1002/cssc.201601235 (2017).

5 Katayama, Y. *et al.* Cloning and expression of *Pseudomonas paucimobilis* SYK-6 genes involved in the degradation of vanillate and protocatechuate in *P. putida*. *Mokuzai Gakkaishi* **33**, 77-79 (1987).

6 Studier, F. W. & Moffatt, B. A. Use of bacteriophage T7 RNA polymerase to direct selective high-level expression of cloned genes. *J. Mol. Biol.* **189**, 113-130 (1986).

7 Bolivar, F. & Backman, K. Plasmids of *Escherichia coli* as cloning vectors. *Methods Enzymol.* **68**, 245-267, doi:10.1016/0076-6879(79)68018-7 (1979).

8 Figurski, D. H. & Helinski, D. R. Replication of an origin-containing derivative of plasmid RK2 dependent on a plasmid function provided in *trans*. *Proc. Natl. Acad. Sci. U. S. A.* **76**, 1648-1652 (1979).

9 Kaczmarczyk, A., Vorholt, J. A. & Francez-Charlot, A. Markerless gene deletion system for Sphingomonads. *Appl. Environ. Microbiol.* **78**, 3774-3777, doi:10.1128/AEM.07347-11 (2012).

10 Kaczmarczyk, A., Vorholt, J. A. & Francez-Charlot, A. Cumate-inducible gene expression system for Sphingomonads and other *alphaproteobacteria*. *Appl. Environ. Microbiol.* **79**, 6795-6802, doi:10.1128/AEM.02296-13 (2013).
